# Maladie de Fabry: à propos d’un cas atypique

**DOI:** 10.11604/pamj.2017.28.291.13388

**Published:** 2017-12-05

**Authors:** Hamid Jallal, Ali Khatori, Laila Bendriss

**Affiliations:** 1 Service de Cardiologie, 3^ème^ Hôpital Militaire Laayoune, Maroc

**Keywords:** Hypertrophie ventriculaire gauche, insuffisance cardiaque, échocardiographie, Left ventricular hypertrophy, heart failure echocardiography

## Abstract

Nous rapportons ici le cas d’un patient âgé de 60 ans présentant une insuffisance cardiaque globale dans le cadre d’une atteinte cardiaque de la maladie de Fabry (MF). Ce cas clinique offre l’opportunité de parcourir la littérature sur l’atteinte cardiaque liée à cette affection ainsi que la particularité de la variante cardiaque.

## Abstract (en)

We here report the case of a 60-year old patient with congestive heart failure due to Fabry disease-related cardiopathy. This study has offered the opportunity to perform a literature review on cardiopathy related to this disease as well as to highlight the features of cardiac manifestation in Fabry disease.

## Introduction

La maladie de Fabry est une pathologie de surcharge lysosomale secondaire à un déficit en α-galactosidase A et de transmission récessive liée à l’X due à des mutations dans le gène codant pour l’alpha-galactosidase A (Xq22.1, plus de 700 mutations décrites). Il s’agit d’une maladie multisystémique touchant préférentiellement le rein, le cœur, la peau, la cornée et le système nerveux. En plus du phénotype classique de la maladie de Fabry, des variants « cardiaques » ont été identifiés, Ces variants cardiaques se caractérisent par la présence d’une cardiomégalie touchant la paroi postérieure du ventricule gauche et le septum interventriculaire, et des atteintes valvulaires associées, mais également par la rareté des manifestations classique de la maladie.

## Patient et observation

Monsieur B., âgé de 60 ans de race noire est adressé au service de cardiologie pour une dyspnée en aggravation, accompagnée d’œdèmes des membres inférieurs et d’ascite, d’asthénie et de palpitations. Il n’a pas de facteurs de risque cardiovasculaires. 7 ans avant son admission, le patient accusait une surdité bilatérale progressive avec des paresthésies des extrémités, compliqué quelques années par la suite d’une hémiparésie droite négligé par le patient et son entourage. Le traitement à l’admission consiste en la prise de furosémide 40 mg (IVD) 3 fois par jour, spironolactone 25mg, cardiensiel 5mg et sintrom 4mg.

A l’examen clinique, le poids est de 90 kg pour une taille de 1,80 m. Le pouls est irrégulier aux environs de 110 battements par minute. La pression artérielle est de 140/70 mmHg. A l’auscultation, les bruits cardiaques sont assourdis et irrégulier avec souffle d’IT, râles sous crépitants de stase. Il existe des œdèmes jusqu’à mollet, prenant le godet, avec ascite et hépatomégalie, reflux hépato-jugulaire. L’examen clinique note par ailleurs une hémiparésie droite et une hypoacousie bilatérale.

**Figure 1:**
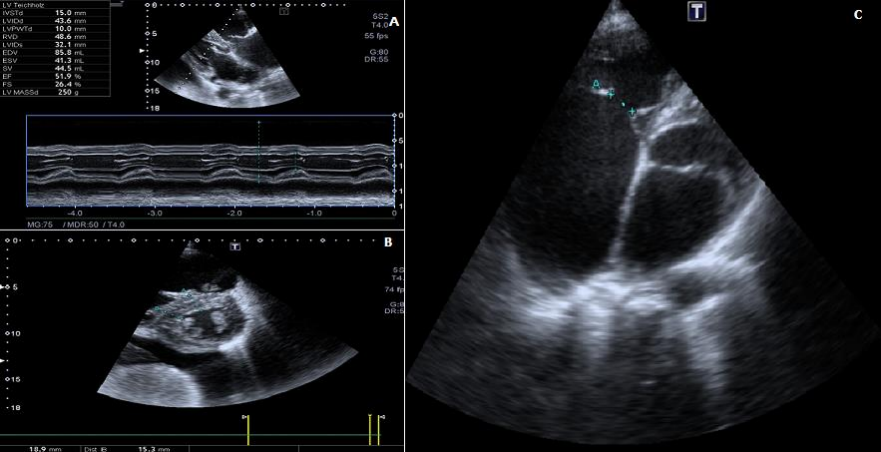
Échocardiographie transthoracique montrant une HVG d’allure sarcomérique (A,B); échocardiographie montrant un hiatus tricuspidien important (C)

L’ECG confirme la présence d’une fibrillation auriculaire avec une réponse ventriculaire rapide associée à un aspect d’ischémie sous-épicardique dans le territoire latéral. La radiographie pulmonaire montre une cardiomégalie avec débord droit et HTAP. TDM cérébral en faveur d’un foyer d’ischémie cérébral ancien. Sur le plan biologique, on notait une insuffisance rénale modérée (clairance de la créatinine à 40 mL/min), une protéinurie minime (0,21 g/24 h), un bilan hépatique perturbée et une élévation du NT-pro-brain natriuretic peptide (NT-proB-NP) à 3 989 ng/L (N < 300 ngL). La troponine-T étaient normal. l La sérologie du virus de l’immunodéficience humaine (VIH) était négative. L’électrophorèse des protéines est normale. L’échocardiographie (Figure 1) retrouvait une fonction systolique ventriculaire gauche normale (FE : 52 %) avec une dilatation du massif atrial surtout droit et une HVG prédominant sur le septum interventriculaire (SIV) mesuré à 16 mm en télèdiastole sans obstruction au doppler.

On note une artialisation du ventricule droit secondaire à une IT importantes sur prolapsus de la valve septale entrainant un hiatus de 14 mm, on note également une IM minime sur une valve épaissie. La fonction diastolique était anormale avec un profil transmitral de type « pseudo-normal » (E/A = 1,2, E/E’ = 15) en faveur d’une élévation des pressions télédiastoliques du ventricule gauche. Il y avait une élévation de la pression artérielle pulmonaire systolique à partir d’un flux d’insuffisance tricuspide laminaire. Il n’y avait pas d’anomalie des autres valves cardiaques ou d’aspect évocateur de fibrose endomyocardique. Enfin, il y avait un épanchement péricardique de moyenne abondance (11 mm) en regard de la paroi postérieure et de l’oreillette droite.

Le diagnostic de maladie de Fabry est évoqué. Un dosage d’alphagalactosidase A au niveau sanguin et urinaire est effectué et revient élevées. Une coronarographie faite dans le cadre du bilan préopératoire en vue de réparer l’insuffisance tricuspidienne est revenue normale. Sur le plan clinique, le patient a évolué défavorablement lors de son retour de la coronarographie avec progression de l’insuffisance rénale et décompensation aigue ayant conduit vers le décès du malade. Aucun traitement spécifique n’a été entrepris chez ce patient.

## Discussion

La MF est une maladie lysosomale de surcharge, par anomalie du métabolisme des glycosphingolipides, liée à l’X et due à des mutations dans le gène codant pour l’alpha-galactosidase A (Xq22.1, plus de 700 mutations décrites). Ces mutations résultent en l’accumulation de globotriaosylcéramide dans toutes les cellules de l’organisme, dont celles de la peau, des reins, du système nerveux et du cœur, entraînant fibrose et dysfonction d’organes, symptômes et décès prématuré [1]. L’homme est en général plus sévèrement affecté que la femme et à un plus jeune âge, les complications cardiovasculaires étant une source importante de morbidité et mortalité. Chez la femme hétérozygote, les symptômes et signes pathologiques apparaissent une dizaine d’années plus tard que chez l’homme, mais l’atteinte cardiovasculaire reste la source principale de morbidité. C’est une cause importante d’hypertrophie ventriculaire non diagnostiquée en l’absence d’hypertension systémique et de sténose aortique. Le développement d’une HVG et d’un remodelage ventriculaire défavorable laisse présager un mauvais pronostic.

On peut schématiquement distinguer 3 phases de la maladie (Tableau 1). La description qui suit concerne les hommes. L’ensemble des symptômes décrits peut également concerner la femme. En général, les symptômes sont décalés dans le temps d’environ 10 ans avec un âge plus tardif d’apparition chez la femme

**Tableau 1:** Chronologie d’apparition et de sévérité des symptômes au cours de la maladie de Fabry

0-20 ans	20-30 ans	Après 30 ans
Acroparesthésies Angio-kératomes Anomalie de la sudation Cornée verticalisée Troubles digestifs	Acroparesthésies Angio-kératomes Anomalie de la sudation Cornée verticalisée Troubles digestifs Protéinurie	Acroparesthésies Angio-kératomes Anomalie de la sudation Cornée verticalisée Troubles digestifs Protéinurie Insuffisance rénale Atteinte neurologique-AVC, baisse de l’audition Atteinte cardiaque

AVC=accident vasculaire cérébrale

Le maître symptôme de la première phase dite phase précoce est la douleur des extrémités, qu’il faut savoir rechercher à l’interrogatoire. La deuxième phase de la maladie concerne les patients âgés de 20 à 30 ans. La plupart des symptômes vus précédemment persistent ou progressent. En revanche, les douleurs peuvent diminuer progressivement et spontanément, voire disparaître. Il est donc très important à cette phase de demander s’il existe des douleurs mais aussi s’il existait des douleurs dans le passé. En général, l’atteinte rénale apparaît à cet âge avec une protéinurie habituellement asymptomatique. Il n’existe en général pas d’hypertension artérielle à ce stade. La troisième phase ou phase tardive de la maladie concerne les patients âgés de plus de 30 ans.

### Diagnostic

La MF peut se présenter sous la forme de variantes cardiaques, c’est-à-dire avec une atteinte cardiaque prédominante, voire exclusive. Ces patients sont en général diagnostiqués plus tard, sur des signes et symptômes cardiaques au premier plan et parfois isolés [2, 3]. Chez les patients avec cardiomyopathie hypertrophique (CMH) diagnostiquée en milieu cardiologique, ces variantes ne sont pas exceptionnelles, avec une prévalence évaluée entre 0,5% dans la population adulte et 1,8% chez les hommes de plus de 40 an. La présentation clinique est parfois celle d’une CMH sarcomérique typique avec hypertrophie septale prédominante, obstruction modérée à sévère et, fréquemment, bloc atrioventriculaire de haut degré nécessitant l’implantation d’un pacemaker. La Société européenne de cardiologie recommande de dépister les signes d’alerte devant évoquer une MF en cas de CMH (Tableau 2) et le dosage systématique de l’activité alpha galactosidase A dans le plasma ou sur globules blancs circulants en cas de diagnostic de CMH chez l’homme de plus de 30 ans et le test génétique chez la femme [4].

**Tableau 2:** Eléments en faveur d’une maladie de Fabry en cas de CMH isolée

Antécédents familiaux compatibles avec une transmission liée à l’X
Antécédents familiaux d’AVC ou d’insuffisance rénale sévère
Anomalie neurosensorielle
Atteinte visuelle
Angio-kératome
PR court
BAV complet
HVG concentrique
Hypokinésie du ventricule gauche
Hypertrophie du ventricule droit
Dysfonction rénale
Protéinurie
Rehaussement tardif dans les zones basales postérolatérales en IRM
Activité circulante alpha-galactosidase A effondré chez l’homme ou génotype positif chez la femme

### Pathophysiologie de la cardiomyopathie conséquente de la MF

Il se produit une accumulation de glycosphingolipide dans les lysosomes du tissu cardiaque, incluant le myocarde, l’endocarde (endothélium) et le système de conduction, où elle est responsable des multiples manifestations CV de la MF [5, 6]. De nouvelles données indiquent qu’il existe une accumulation extralysosomale considérable de globotriaosylcéramide (Gb 3). Deux événements pathophysiologiques importants sont liés aux lésions directes résultant de l’accumulation de glycosphingolipide stocké et à l’hypothèse de la déplétion énergétique, selon laquelle le glycosphingolipide a des effets secondaires sur le métabolisme cardiaque. Pratiquement, la cardiomyopathie classique de cette maladie est liée à une hypertrophie compensatoire consécutive à la mort cellulaire et à la déplétion en énergie avec surrégulation de la vacuolisation autophagique et de l’apoptose, inflammation, dysfonction endothéliale et perte de contrôle par le système nerveux autonome. Ce qui se traduit: au niveau myocardique par une cardiomyopathie hypertrophique; au niveau endocardique, par des altérations valvulaires le plus souvent mitrales et aortiques; au niveau endothélial par un syndrome coronarien; au niveau de la conduction, par des arythmies complexes; au niveau du SN autonome, par une réduction de la variabilité du rythme.

L’autre particularité est la présence du Gb3, le métabolite « coupable » dans d’autres organelles que le lysosome. Cette accumulation se traduit notamment au niveau endothélial par un rétrécissement extérieur aux artères coronaires, marqué surtout sur les petites artères, ce qui nécessitera une prise en charge différente de l’atteinte classique des artères de plus gros calibre. A un stade plus tardif, une désorganisation fibreuse suivie d’une fibrose irréversible marque également la maladie [7]. Pour expliquer cette hypertrophie des myocytes, la déplétion énergétique liée à l’inefficience de l’utilisation de l’ATP est une hypothèse alléchante, d’autant plus que l’enzymothérapie substitutive engendre une réduction de volume des vacuoles [7]. Elle va de pair avec une réduction de la contractilité cardiaque, une insuffisance mitochondriale et une apoptose qui toutes mènent à l’hypertrophie. « Dans ce cadre, on comprend mieux qu’une enzymothérapie précoce puisse normaliser les cellules cardiaques en 6-8 semaines ».

### Manifestations cardiovasculaires de la MF

Bien que les manifestations cliniques puissent être hétérogènes, dans sa forme classique, la MF a une évolution lente. L’atteinte cardiaque est fréquente et peut être variable. Cependant, la MF est une cause importante de cardiopathie avec HVG et un profil de remplissage restrictif. L’atteinte des différentes parties du cœur entraîne différentes altérations du système CV et dans l’ensemble, les patients développent une cardiomyopathie hypertrophique infiltrative évolutive, des arythmies, des anomalies de la conduction, une coronaropathie et/ou des anomalies valvulaires [8]. L’atteinte cardiaque peut être l’unique symptôme chez certains sujets masculins hémozygotes et jusqu’à 5% de sujets masculins et 12% de sujets féminins atteints de cardiomyopathie hypertrophique d’apparition tardive peuvent présenter la variante cardiaque de la MF [9, 10]. La dysfonction diastolique est une caractéristique fréquente de cette variante. Par opposition aux cardiomyopathies restrictives réelles, une physiologie restrictive est rarement observée mais si elle est présente, elle survient le plus souvent à un stade avancé de la maladie et est associée à une fibrose importante. Le syndrome d’HVG, de fibrose interstitielle et de vasculopathie métabolique entraîne une dysfonction diastolique avec une susceptibilité accrue à l’insuffisance cardiaque diastolique. Si l’endocarde est touché, il y a une détérioration progressive des valves mitrales et aortiques (épaississement des feuillets valvulaires) avec le développement d’un prolapsus de la valve mitrale et une régurgitation mitrale légère, l’atteinte des valves aortique ou tricuspidienne est plus rare mais réelle comme on vient de le voir chez notre patient. Lorsque l’endothélium des artères coronaires est atteint, une coronaropathie progressive se développe (et une ischémie myocardique) sans sténose luminale des artères coronaires épicarpiques. Une atteinte directe du tissu de conduction et du système nerveux autonome peut entraîner des troubles de la conduction et des bradyarythmies, alors que l’hypertrophie ventriculaire et la fibrose peuvent augmenter la sensibilité aux tachyarythmies ventriculaires [11].

### Traitement

L’avènement de la thérapie enzymatique substitutive (TES) pour les troubles lysosomaux, incluant la MF, a révolutionné nos connaissances sur ces maladies et a eu des effets très bénéfiques pour les patients. La TES a ralenti l’évolution de la néphropathie et a amélioré la douleur neuropathique chez les patients atteints de la MF [12]. La TES vise à arrêter l’évolution de l’atteinte des organes cibles, y compris des manifestations cardiaques et à améliorer les symptômes cliniques de la MF [12]. C’est lorsque le traitement est institué à un stade précoce de la maladie que les bénéfices de l’enzymothérapie substitutive sont les plus importants. Cela est malheureusement rarement le cas en raison du délai diagnostique et de prise en charge (plus de 10 ans par exemple pour les variantes cardiaques).

### Traitement symptomatique

La prise en charge des complications cardiovasculaires obéit d’abord aux recommandations habituelles concernant l’hypertension artérielle et les autres facteurs de risque, l’insuffisance cardiaque et les troubles du rythme; IEC (ou sartans) et statines sont largement prescrits chez ces malades présentant souvent une atteinte rénale. Quelques particularités doivent cependant être connues: En cas d’arythmie atriale, même non soutenue ou rare, le traitement anticoagulant oral à vie est la règle (le score CHA2DS2-VASc ne s’applique pas dans ce cas, le risque thromboembolique est toujours élevé); En cas d’incompétence chronotrope symptomatique, les indications de pacemaker ne sont pas illicites; La fréquence élevée des troubles conductifs et des arythmies, comme de l’insuffisance cardiaque devraient amener à élargir les indications de pacemaker et de resynchronisation cardiaque (avec ou sans défibrillateur); Pour les raisons précédentes, les bradycardisants et notamment les bêtabloqueurs peuvent être mal tolérés, particulièrement chez les patients avec QRS large, dysfonction systolique ventriculaire gauche et/ou insuffisance rénale sévère; L’amiodarone empêche (in vitro) l’action de l’enzymothérapie substitutive et devrait être évitée au long cours.

### Substitution enzymatique

Deux types de traitement par remplacement sont utilisables: l’agalsidase beta (fabrazyme^®^). L’agalsidase alpha (replagal^®^). Bien que ces produits soient fonctionnellement équivalents à la même posologie [13, 14], les essais ont été conduits à des doses différentes: 1mg/Kg toutes les 2 semaines pour la forme beta; 0.2mg/Kg une semaine sur deux pour la forme alpha. Les résultats des études contrôlées en phase II de la forme alpha-agalsidase ont été une amélioration des douleurs par rapport au placebo, ainsi qu’une amélioration des anomalies rénales et une amélioration de la conduction cardiaque. La forme bêta a fait disparaitre les dépôts microvasculaires endothéliaux; une étude européenne a montré la stabilisation de la fonction cardiaque [15] et du fonctionnement nerveux périphérique avec réduction nette des douleurs.

## Conclusion

La maladie de Fabry est une maladie importante à connaître car elle bénéficie d’un traitement spécifique, elle est causée par un défaut métabolique héréditaire lié au chromosome X de l’enzyme lysosomale a-Gal A, qui entraîne un stockage lysosomal anormal. Les patients présentant la variante d’apparition tardive de la MF sont atteints d’insuffisance cardiaque, d’arythmies et de valvulopathie, notre cas clinique sort de l’habitudes et montre que la valve tricuspide peut être sévèrement touchée à l’instar des autres atteintes valvulaires gauche décrites dans la littérature. Il est donc capital, devant une CMH d’allure sarcomérique, d’évoquer le diagnostic et de rechercher des signes qui vont orienter vers un variant cardiaque de maladie de Fabry, Bien que la mesure de l’a-galactosidase plasmatique ait une valeur pronostique, une analyse génétique fournit une confirmation diagnostique. L’enzymothérapie reste la clé de voûte de la prise en charge, à condition qu’elle soit instituée précocement.

## Conflits d’intérêts

Les auteurs ne déclarent aucun conflit d'intérêts.
